# Incidence and Risk Factors for Atrial Fibrillation Recurrence after Ablation of Nodal and Atrioventricular Reentrant Tachycardia: A Meta-analysis

**DOI:** 10.7759/cureus.7824

**Published:** 2020-04-25

**Authors:** Estelle Torbey, Boutros Karam, Elsa Sleiman, Rabih Tabet, Malcolm Kirk, David Donaldson, Antony D Chu

**Affiliations:** 1 Electrophysiology, Rhode Island University Hospital - Warren Alpert Medical School of Brown University, Rhode Island, USA; 2 Cardiology, Staten Island University Hospital - Northwell Health, Staten Island, USA; 3 Internal Medicine, Staten Island University Hospital - Northwell Health, Staten Island, USA; 4 Cardiovascular Medicine, Staten Island University Hospital - Northwell Health, Staten Island, USA; 5 Cardiology, Rhode Island University Hospital - Warren Alpert Medical School of Brown University, Rhode Island, USA

**Keywords:** atrial fibrillation, atrio-ventricular nodal re-entrant tachycardia, atrio-ventricular re-entrant tachycardia, catheter ablation, recurrence predictors

## Abstract

Introduction

Atrioventricular nodal reentrant tachycardia (AVNRT) and atrioventricular reentrant tachycardia (AVRT) are frequently associated with atrial fibrillation (AF). Targeting the slow or accessory pathways has been advocated as therapy for coexisting AF. But in practice, AF has frequently recurred after ablation, possibly because of various risk factors. The objective of this study is to investigate these risk factors and check for their significance in AF recurrence.

Materials and methods

A systematic review of Medline, Cochrane, and ClinicalTrials.gov databases was conducted. Articles that studied AF recurrence after either AVNRT or AVRT ablation were reviewed. Publication bias was adequately assessed, and the random method was applied for all dichotomous values. Finally, the odds ratio (OR) and confidence intervals (CI) were calculated for each risk factor.

Results

Four studies were included, with a total of 1,308 participants. Only 218 participants had dual tachycardia (AF with either AVNRT or AVRT). The mean follow-up time was 29 +/- 3.3 months. The mean age was 56 +/- 15 years. Age constituted the only significant risk factor for AF recurrence (OR: 3.4, CI: 2.1-5.3, p<0.001). Atrial vulnerability did not significantly correlate with a higher risk of AF recurrence (OR: 4.8, CI: 0.7-29, p<0.008). Again, neither male gender (OR: 1.5, CI: 0.8-2.8, p<0.16) nor left atrial diameter (OR: 1.5, CI: 0.2-10, p<0.67) were significant risk factors for recurrence of AF.

Conclusion

Older age was the only significant predictor of AF recurrence after ablation of AVNRT or AVRT. Further studies are needed to determine the age cut-off at which concomitant pulmonary vein isolation would be beneficial in patients undergoing ablation of AVNRT/AVRT.

## Introduction

Supraventricular tachycardia (SVT), such as atrioventricular nodal reentrant tachycardia (AVNRT) and atrioventricular reentrant tachycardia (AVRT), is frequently associated with atrial fibrillation (AF) [1]. The prevalence of AF in these patients was noted to be higher than the general population, yet for unknown reasons. Possible explanations include enhanced atrial vulnerability, and degeneration of SVT into AF [2]. Although the specific role of the accessory or the slow pathways in the pathogenesis of AF is not clear, surgical or catheter ablation of these pathways often reduces the episodes of spontaneous AF. However, 9%-28% of patients who underwent successful catheter ablation of an accessory pathway or slow pathway modification had a recurrence of AF [3]. Persistent atrial vulnerability post-ablation was demonstrated as a risk factor for recurrence of AF in previous studies, as well as the patient’s age and the left atrial diameter.

This study has three main goals. First, we analyzed the effect of atrial vulnerability on the recurrence of AF in patients with previously ablated SVT and a history of AF. Second, we determined the incidence of AF before and after SVT ablation. And finally, we determined the risk factor(s) affecting the recurrence of AF after ablation of an accessory pathway or slow pathway modification.

## Materials and methods

A literature search was independently performed by our investigators. The electronic databases of Medline, Cochrane, and ClinicalTrials.gov were searched up until September 2017. The following searching terms were adopted: [AVNRT] OR [AVRT], OR [Atrial vulnerability] OR [Atrial Fibrillation], AND [Recurrence] OR [Accessory Pathway].

The random method was applied for all dichotomous values to derive an overall summary estimate for each risk factor. Heterogeneity was quantified using the I^2^ statistic test. Publication bias was assessed using funnel plots. Unadjusted studies did not adjust for any potential confounders and were excluded from the analysis. Studies that adjusted only for age were considered as minimally-adjusted and therefore were excluded in a sensitivity analysis. Finally, adequately-adjusted studies adjusted for age, gender, and at least two established cardiovascular risk factors were included in the analysis. Odds ratio (OR) and confidence intervals (CI) were assessed for each risk factor.

Inclusion criteria

We included studies done on patients aging 18 or more. Retrospective, as well as prospective studies, were included. Both randomized and non-randomized controlled trials were counted in because of the scarcity of randomized studies that fit the inclusion criteria. The population studies with the following inclusion criteria were included in the meta-analysis: documented regular arrhythmia (AVNRT or AVRT) with a history of spontaneous AF prior to ablation were included; the coexisting pathway could have been either concealed or manifest. A mandatory follow-up period of more than a year was required with regular clinic visits, Holter, or event monitor. Patients should not be taking anti-arrhythmic medications, post-SVT ablation. Studies with data on atrial vulnerability defined as the induction of AF for more than 30 seconds with one or two extra stimuli were included in the analysis. And finally, the recurrence of AF should be mentioned as a clinical endpoint.

Exclusion criteria

On the other hand, studies that comprised patients with congenital structural heart disease were excluded from the meta-analysis. Also were excluded studies that included patients with supraventricular arrhythmia other than AVRT or AVNRT (e.g., atrial tachycardia). Studies in which the population did not have documented the previous episodes of atrial fibrillation were excluded along with those that had a follow-up post-ablation less than one year or a concomitant recurrence of the SVT occurred along with the recurrence of AF. Finally, we excluded the studies that did not specify risk ratios, or from which a risk ratio could not be calculated.

Data were extracted using a standardized form in duplicate. General study characteristics, including the population under study, number of participants with and without recurrence of AF, duration of follow-up, mean age, number of men, and number of participants with atrial fibrillation at baseline, were extracted. Maximally adjusted measures of relative risk and associated 95% confidence intervals for outcomes of interest (atrial fibrillation recurrence) were extracted, as well as the published covariates that investigators included in the regression model such as age, left atrial diameter, atrial vulnerability, and male gender. The extracted data were analyzed using the SPSS version 24 software package (IBM Inc., Armonk, US).

The abstract of this article was previously presented at the Heart Rhythm Society (HRS) scientific sessions 2017 in Chicago, Illinois.

## Results

**Figure 1:**
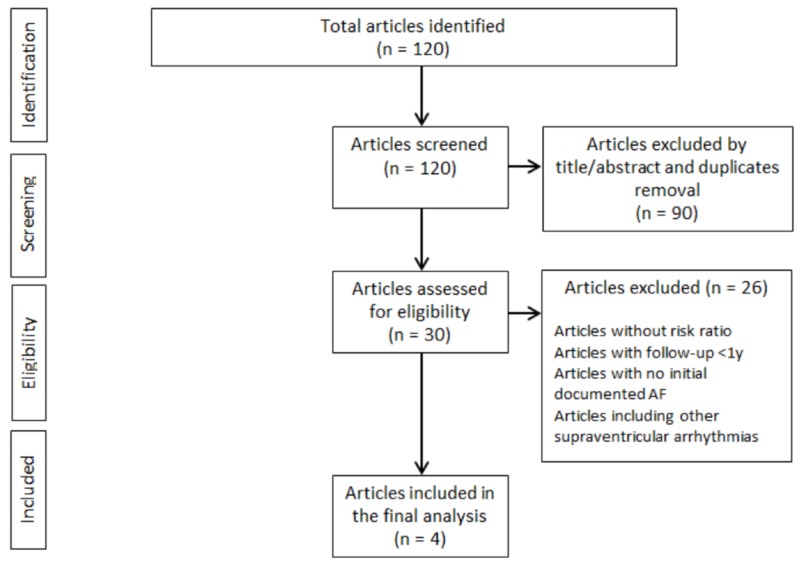
Studies selection flow chart AF - atrial fibrillation

A total of 120 abstracts were reviewed. Only 30 articles met the primary eligibility criteria. After reviewing all 30 articles, only four studies were included in the final analysis (Figure 1).

Baseline characteristics of the patients included in all four studies are shown in the table below (Table 1).

**Table 1:** Baseline characteristics of patients included in all four studies in the meta-analysis AVNRT - atrioventricular nodal reentry tachycardia; AVRT - atrioventricular reentry tachycardia; AF - atrial fibrillation; SVT - supraventricular tachycardia (a term used to include both AVRT and AVNRT); DM - diabetes mellitus; HTN - hypertension; EF - ejection fraction; AP - accessory pathway; HR - heart rate

Study	Total (n)	AVNRT (n)	AVRT (n)	AF prior to ablation (n)	Incidence of AF after ablation (n)			Mean follow-up (months)	Ablated structure	Male sex (AF+SVT group)	Mean age			Atrial vulnerability difference between AF recurrence and no AF recurrence group	Left atrial diameter (> 40mm) difference between AF recurrence and no AF recurrence group	Other risk factors studied
					In patients with prior AF	In patients without prior AF	In total				AF + SVT group (prior to ablation)	AF recurrence group	No AF recurrence group			
Dagres et al. (2001) [4]	191	-	191	91/191 (47%)	18/91 (20%)	4/100 (4%) (p<0.01)	22/191 (11%)	23 +/- 12	Accessory pathway	60%	42 +/- 15	50 +/- 12	40 +/- 16 (p=0.008)	p=0.44	-	Structural cardiac disease, multiple pathways, pathway location
Oddsson et al. (2002) [5]	183	-	183	54/183 (29%)	13/54 (24%)	0/129 (0%)	13/183 (7%)	24 +/- 12	Accessory pathway	74%	45 +/- 15	53 +/- 13	42 +/- 15 (p=0.0001)	-	-	Years in SVT, pre-excitation, atrial vulnerability
Amasyali et al. (2005) [6]	533	533	-	36/533 (6%)	10/36 (28%)	2/497 (0.4%) (p<0.01)	12/533 (2%)	34 +/- 11	Slow pathway	60%	49 +/- 17	56 +/- 16	46 +/- 16 (non-significant p-value)	p<0.007	p=0.002	Valvular disease, DM, HTN
Wang et al. (2005) [7]	401	-	401	37/401 (9%)	4/36 (11%)	2/364 (0.5%)	6/401 (1%)	36 +/- 11	Accessory pathway	30%	43 +/- 14	64 +/- 5	40 +/- 11 (p=0.01)	p=0.01	Non-significant p-value	EF, antegrade v/s retrograde AP, years in tachycardia, HR

**Figure 2:**
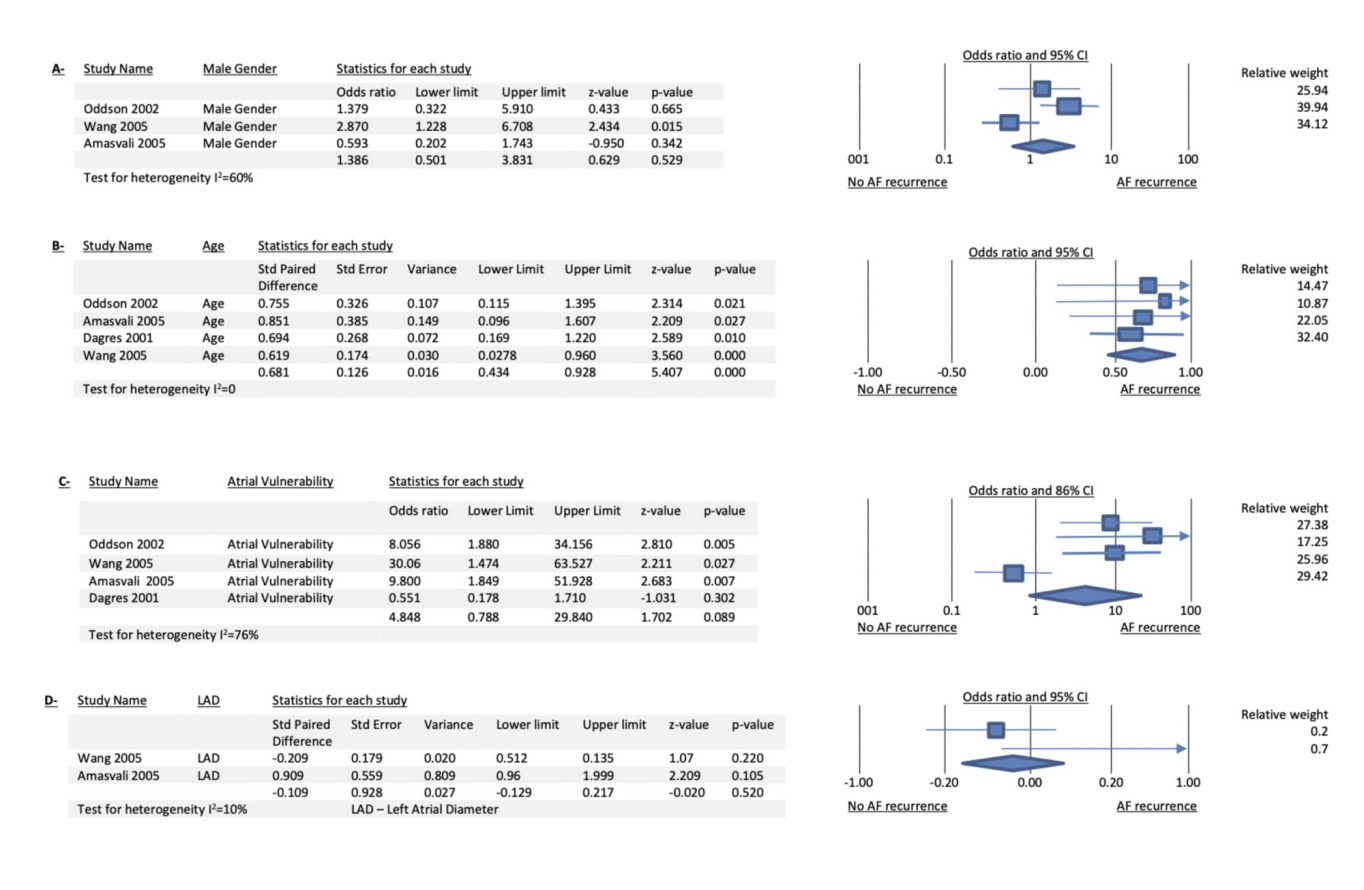
Combined forest plots for different risk factors studied Panels A, B, C, and D show the forest plots for the different risk factors studied (male gender, age, atrial vulnerability, and left atrial diameter respectively). LAD - left atrial diameter; AF - atrial fibrillation; CI - confidence interval

Among 1,308 patients with reentrant tachycardia (either AVRT or AVNRT), AF occurred in 16.66% of patients prior to the ablation procedure. Among 218 patients with dual tachycardia (AF with either AVRT or AVNRT) who underwent successful ablation of their accessory pathway or modification of the slow pathway without recurrence of their respective regular tachycardia, 45 (20%) had AF recurrence. The mean age of these patients with AF recurrence was 56 +/- 15 years. There was a relative risk reduction of 81% for AF recurrence after SVT ablation over a mean follow-up of 29 +/- 3.3 months with serial Holters or clinic visits. Age was the only significant risk factor for AF recurrence (OR: 3.4, CI: 2.1-5.3, p<0.001). Atrial vulnerability did not correlate with a higher risk of AF recurrence (OR: 4.8, CI: 0.7-29, p<0.008). Also, neither male gender (OR: 1.5, CI: 0.8-2.8, p<0.16) nor left atrial diameter (OR: 1.5, CI: 0.2-10, p<0.67) were significant risk factors for recurrence of AF after either slow pathway modification or accessory pathway ablation (Figure 2).

## Discussion

This article constitutes the first meta-analysis to investigate the risk factors and outcome of recurrence of AF after ablation of a large number of patients with AVRT/AVNRT. Previous studies on the mechanism of AF in patients with AVRT or AVNRT failed to elicit a specific explanation, and the role of the accessory or slow pathway in the pathogenesis of AF remains unclear. It has been postulated that the increased risk of AF in these patients was due to branching causing micro-reentrant atrial circuits, dispersion of atrial refractoriness, decreased atrial cycle length with an increased sympathetic tone, and atrial stretch, and atrial cellular hypoxia during fast reentrant tachycardia degenerating into atrial fibrillation [4-8]. Also, the ligament of Marshall muscle bundles have been implicated in both triggering of AF (along with the ganglionated plexi present in and around the ligament) as well as taking part in accessory pathway conduction in patients with pre-excitation syndrome [9].

Theoretically, if the accessory/slow pathway was the only contributor to clinical AF in this population, then, ablation of this pathway would have prevented AF in all patients. But, in all the studies that were included in this meta-analysis, the risk of AF recurrence after ablation was only reduced but not completely eliminated, with a mean recurrence rate of 20%. This implies that the mechanism of AF in patients with either AVRT or AVNRT is much more complex, and the accessory/slow pathways constitute only one part of the many potential causes of AF.

Because AF is associated with significant mortality and morbidity that can be markedly lessened by means of pharmacological intervention, timely and appropriate identification of patients at high risk of recurrence of AF after successful catheter ablation is of major importance. In previous studies, many risk factors, such as atrial vulnerability, age, left atrial diameter, and structural heart disease seemed to entertain the persistence of AF after successful ablation of reentrant tachycardia [4-7]. In our study, we investigated the relationship between AF recurrence and the main factors that were found to have a significant impact on all the previous studies. Among these risk factors, only age constituted a significant and independent predictor of AF recurrence, after taking into account the other variables. Atrial vulnerability tends to be higher in patients who had a recurrence of AF but did not reach statistical significance when all the studies were combined together. Finally, sex and left atrial diameter had no statistically significant impact on AF recurrence after ablation in this population. These results are most likely due to additional age-related atrial structural remodeling that is independent from the electro-anatomic implications and correlations related to the reentrant tachycardia. These data suggest that older patients, after catheter ablation of AVRT/AVNRT, are at increased risk of AF recurrence and should be followed-up closely, or even have a pulmonary vein isolation procedure done at the same time of ablation.

Few limitations can be noted in our study. First, it included both randomized and non-randomized controlled trials as well as both retrospective and prospective studies. Second, the narrow and specific inclusion/exclusion criteria that we set limited the number of included studies in the final analysis to a total of four articles only, with a total number of 1,308 patients. This might have affected the power of our meta-analysis since many studies were excluded because they lacked some of the data that we required for our analysis. Finally, the incidence of AF recurrence may have been underestimated because most patients were followed by clinic visits and Holter monitoring post-ablation and not with implantable loop recorders.

## Conclusions

This study is the first meta-analysis done on the largest group of patients with AVRT/AVNRT and AF. It showed that the arrhythmogenic substrate for AF does not exactly correlate with the substrate for the reentrant tachycardias in patients with dual tachycardia. Curing patients from reentrant tachycardia does not totally eliminate the risk of recurrent AF, especially in older patients who will eventually require closer follow-up and likely pulmonary vein isolation.
